# Lung fibroblasts express a miR-19a-19b-20a sub-cluster to suppress TGF-β-associated fibroblast activation in murine pulmonary fibrosis

**DOI:** 10.1038/s41598-018-34839-0

**Published:** 2018-11-09

**Authors:** Kunihiko Souma, Shigeyuki Shichino, Shinichi Hashimoto, Satoshi Ueha, Tatsuya Tsukui, Takuya Nakajima, Hiroshi I. Suzuki, Francis H. W. Shand, Yutaka Inagaki, Takahide Nagase, Kouji Matsushima

**Affiliations:** 1 Department of Molecular Preventive Medicine, Graduate School of Medicine, The University of Tokyo, Tokyo, Japan; 2 Department of Respiratory Medicine, Graduate School of Medicine, The University of Tokyo, Tokyo, Japan; 3 Division of Molecular Regulation of Inflammatory and Immune Diseases, Research Institute of Biomedical Sciences, Tokyo University of Science, Chiba, Japan; 4 Department of integrative Medicine for Longevity, Graduate School of Medical Sciences, Kanazawa University, Ishikawa, Japan; 5 David H. Koch Institute for Integrative Cancer Research, Massachusetts Institute of Technology, Cambridge, Massachusetts USA; 6 Center for Matrix Biology and Medicine, Graduate School of Medicine, Tokai University, Kanagawa, Japan

## Abstract

Lung fibroblasts play a pivotal role in pulmonary fibrosis, a devastating lung disease, by producing extracellular matrix. MicroRNAs (miRNAs) suppress numerous genes post-transcriptionally; however, the roles of miRNAs in activated fibroblasts in fibrotic lungs remain poorly understood. To elucidate these roles, we performed global miRNA-expression profiling of fibroblasts from bleomycin- and silica-induced fibrotic lungs and investigated the functions of miRNAs in activated lung fibroblasts. Clustering analysis of global miRNA-expression data identified miRNA signatures exhibiting increased expression during fibrosis progression. Among these signatures, we found that a miR-19a-19b-20a sub-cluster suppressed TGF-β-induced activation of fibroblasts *in vitro*. Moreover, to elucidate whether fibroblast-specific intervention against the sub-cluster modulates pathogenic activation of fibroblasts in fibrotic lungs, we intratracheally transferred the sub-cluster-overexpressing fibroblasts into bleomycin-treated lungs. Global transcriptome analysis of the intratracheally transferred fibroblasts revealed that the sub-cluster not only downregulated expression of TGF-β-associated pro-fibrotic genes, including *Acta*2, *Col1a1*, *Ctgf*, and *Serpine1*, but also upregulated expression of the anti-fibrotic genes *Dcn*, *Igfbp5*, and *Mmp3* in activated lung fibroblasts. Collectively, these findings indicated that upregulation of the miR-19a-19b-20a sub-cluster expression in lung fibroblasts counteracted TGF-β-associated pathogenic activation of fibroblasts in murine pulmonary fibrosis.

## Introduction

Idiopathic pulmonary fibrosis (IPF) is a chronic, progressive, and irreversible type of idiopathic interstitial pneumonia. Due to a lack of effective therapies, the median survival for IPF is from 2.5 to 3.5 years after diagnosis^1^. Currently, lung transplantation is the most effective treatment for IPF, but is associated with serious side effects, such as immune rejection of the graft and increased susceptibility to infection due to immunosuppressive agents.

Fibrosis results from inappropriate wound-healing processes involving repetitive lung injury, inflammation, and exaggerated deposition of extracellular matrix (ECM), such as type I collagen (Col1), leading to the destruction of tissue architecture. Activated fibroblasts, referred to as α-smooth muscle actin (α-SMA)-expressing myofibroblasts, are the major producers of Col1 in fibrotic tissues^2^. Transforming growth factor-beta (TGF-β) plays pivotal roles in fibroblast activation by stimulating TGF-β receptor, resulting in the expression of pro-fibrotic genes, such as connective-tissue growth factor (CTGF)^3^. Platelet-derived growth factor (PDGF) receptor signaling also contributes to fibroblast activation through cell migration and proliferation^4^. Accordingly, regulation of lung fibroblast activation by modulating these signaling pathways represents an attractive therapeutic strategy against pulmonary fibrosis.

MicroRNAs (miRNAs) are a class of small endogenous noncoding RNAs of approximately 22 nucleotides in length. miRNAs negatively regulate the expression of target genes mainly by binding to the 3′ untranslated region (UTR) of the gene, thereby modulating various biological processes^5^. In humans, >45,000 miRNA-target sites have been identified, with miRNAs targeting >60% of protein-coding genes^6^. miRNAs have been implicated in a range of diseases, including cancer^7^, degenerative neurological disease^8^ and inflammatory disease^9^.

A number of miRNAs have been implicated in IPF and pulmonary fibrosis models, including the miR-17~92 cluster^10^, miR-19a,b^11^, miR-21^12^, miR-26a,b^13,14^, miR-29^15^, miR-145^16^, miR-155^17^, and let-7d^18^. In rat models of pulmonary fibrosis, miRNA-expression profiling and proteomics studies suggest that miRNAs influence the proliferative, migratory, invasive, and survival properties of lung cells in the early phase of the disease^19^. However, because most previous studies analyzed miRNAs expressed in whole-lung tissue, the dynamics and roles of miRNAs in fibroblasts of fibrotic lung remain elusive.

In this study, we performed global miRNA-expression profiling of lung fibroblasts during bleomycin-induced transient pulmonary fibrosis and silica-induced progressive pulmonary fibrosis using Col1(α)2-green fluorescent protein (*Col1a2*-GFP)-reporter mice to specifically isolate fibroblasts directly from mouse lungs. We identified the miR-19a-19b-20a sub-cluster, a member of fibrosis-associated miRNA signatures, as a suppressor of TGF-β-associated lung fibroblast activation *in vitro*. Pathway analysis of target genes associated with miRNA signatures also suggested a broad contribution of the miR-19a-19b-20a sub-cluster in TGF-β signaling.

## Results

### Identification of pulmonary fibrosis-associated miRNA signatures by global miRNA-expression profiling of lung fibroblasts

**Figure 1:**
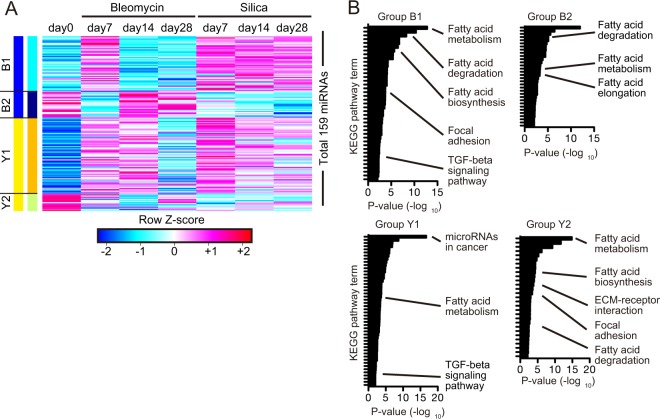
Global expression profile of miRNAs in the activated lung fibroblasts of multiple lung fibrosis models. (**A**) Global miRNA-expression profile of fibroblasts from bleomycin- and silica-treated murine lungs on days 7, 14, and 28, as well as those of untreated mice on day 0. The heat map represents the group of differentially expressed miRNAs determined based on the WGCNA and CLICK methods. Each column represents the group and time point, and each row represents an individual miRNA. (**B**) KEGG pathway enrichment analysis of target genes associated with the miRNA signatures. The enrichment significance of the KEGG pathways was calculated using DIANA-miRPath version 3.0 with Tarbase version 7.0. *P*-values were calculated for each term using a two-sided hypergeometric test with Benjamini-Hochberg correction. See also Supplemental Tables S3 and S4.

To measure global miRNA-expression patterns in activated lung fibroblasts, we performed global miRNA-expression profiling on fibroblasts isolated from untreated, bleomycin-treated, and silica-treated mice on days 7, 14, and 28 post-treatment. We identified 165 miRNAs that were differentially expressed with a fold-change ≥2 between at least two time points. These miRNAs were grouped based on their expression kinetics using the WGCNA and CLICK methods^20,21^ (Fig. 1A; see Supplemental Table S3 for the full list of miRNAs in each group). Within the four miRNA groups identified, groups B1 and Y1 were transiently upregulated and groups B2 and Y2 were transiently downregulated on day 7 in bleomycin model (Fig. 1A). Notably, these expression changes were maintained in the silica-induced chronic pulmonary fibrosis model until at least day 28 post-administration, and no miRNA specifically expressed in the late phase of silica-induced chronic pulmonary fibrosis, was found (Fig. 1A). These results suggest that these miRNAs are associated with pulmonary fibrosis progression independent of the experimental model.

To determine the biological functions associated with these miRNA signatures, we performed KEGG pathway analysis of predicted miRNA-target genes (see Supplemental Table S4 for the full list of significantly enriched KEGG pathways for each group). We found that genes associated with the TGF-β-signaling pathway were enriched in the predicted targets of miRNAs in groups B1 and Y1, but not in groups B2 and Y2 (Fig. 1B). By contrast, ECM-receptor interactions were only associated with group Y2. Because miRNAs are post-transcriptional gene repressors, these results indicated that the miRNAs in groups B1 and Y1 might suppress TGF-β-signaling-pathway related genes in fibrotic lung fibroblasts, and that the miRNAs in group Y2 might suppress ECM-receptor-interaction-related genes. Interestingly, fatty acid metabolism-related pathways (fatty acid metabolism, degradation, biosynthesis, and elongation) were highly enriched in all of the miRNA signatures (Fig. 1B).

### miR-19a-19b-20a sub-cluster is associated with TGF-β-signaling pathway

**Figure 2:**
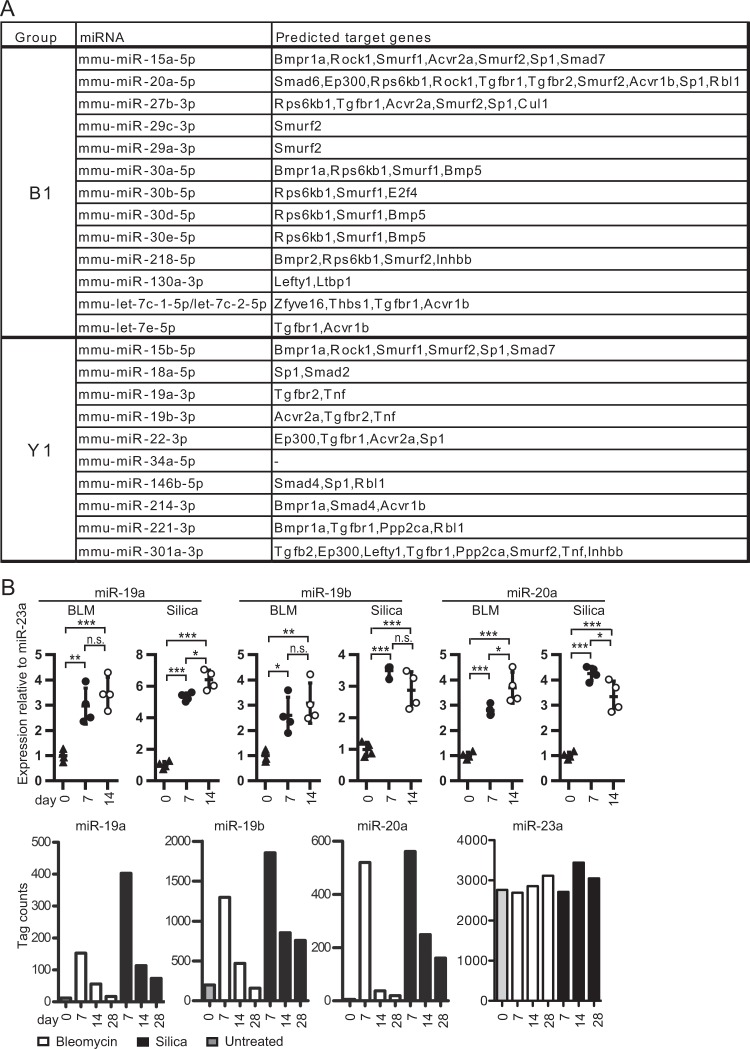
The miR-19a-19b-20a sub-cluster is associated with a part of TGF-β-signaling-associated genes. (**A**) TGF-β-signaling-pathway associated miRNAs in groups B1 and Y1 identified using DIANA-miRPath version 3.0 with Tarbase version 7.0. The number of TGF-β-signaling genes associated with each mature miRNA is shown. (**B**) qPCR validations of miR-19a, 19b and 20a expression in fibroblasts from bleomycin- and silica-treated murine lungs on days 7 and 14, as well as untreated mice on day 0. The relative changes in gene expression were analyzed by the 2^−ΔΔCT^ method. qPCR and global miRNA-expression data showed similar trends related to expression kinetics. Graphs of the qPCR data show the mean ± SEM (*n* = 4). *P < 0.05; **P < 0.01; ***P < 0.001, one-way ANOVA with Tukey-Kramer’s multiple-comparison post hoc test. Cohen’s effect size d in these data was >0.7. (**C**) Details of the TGF-β signaling pathway-related genes potentially targeted with mmu-miR-19a-3p, 19b-3p, 20a-5p and 20a-3p.

Within groups B1 and Y1, we selected miRNAs with mean tag count higher than 100 in the global miRNA-expression profile, and chose the guide strands of either 5p or 3p from these miRNAs. Next, we analyzed the predicted target genes, associated with the TGF-β-signaling pathway, and whose predicted scores had been described in TarBase version 7.0. We found 23 miRNAs with relatively high expression and predicted TGF-β-associated target genes with scores were more than 0.5 (Fig. 2A). Among them, miR-19a, 19b, and 20a formed a sub-cluster, within 400 bp of genomic region. Consistent with the global miRNA-expression profile, qPCR revealed a significant increase of miR-19a, 19b, and 20a-expression in activated fibroblasts in the early phase of bleomycin and silica models (Fig. 2B). Interestingly, miR-19a-19b-20a sub-cluster, as a whole, had 12 predicted TGF-β-associated target genes, each miRNA predicted to target Tgfbr2, a cell surface receptor of TGF-β1. Therefore, we focused on miR-19a-19b-20a sub-cluster as a candidate that regulates fibroblast activation by suppressing TGF-β-signaling.

### miR-19a-19b-20a sub-cluster directly suppresses Tgfbr2 protein expression in primary lung fibroblasts

**Figure 3:**
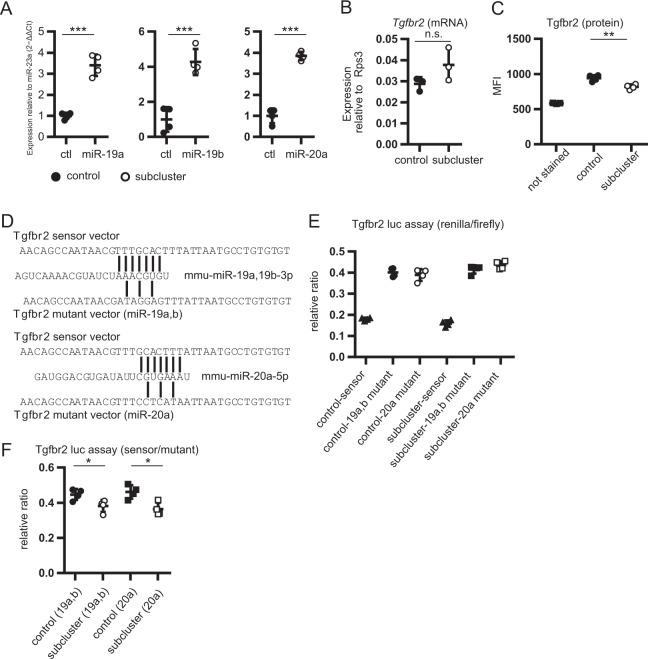
The miR-19a-19b-20a sub-cluster suppresses TGFBR2 protein expression post-transcriptionally by targeting the *Tgfbr2* 3′ UTR sequence in primary lung fibroblasts. (**A**) miR-19a, 19b and 20a expression measured in hCD271^+^ fibroblasts transduced with miR-19a-19b-20a sub-cluster vector was higher than that observed in hCD271^+^ fibroblasts transduced with control vector according to qPCR. (**B**) Tgfbr2 mRNA-expression levels in transduced fibroblasts analyzed by qPCR. (**C**) TGFBR2 protein levels in the transduced fibroblasts analyzed based on MFI. Cells were stained with PE-conjugated anti-hCD271 and APC-conjugated anti-TGFBR2 antibodies. The MFIs of APC conjugates in hCD271^+^ cells were measured by flow cytometry. (**D**–**F**) mmu-miR-19a-3p, 19b-3p and 20a-5p directly targets *Tgfbr2* 3′ UTR. (**D**) Diagram of the complementarity between each miRNAs, the wild-type *Tgfbr2* 3′ UTR, and the each mutant *Tgfbr2* 3′ UTR sequences. (**E**) Luciferase-reporter analysis of the interaction between each miRNAs and the *Tgfbr2* 3′ UTR. The each of control/ sub-cluster-expression vector, and each of the sensor/mutant vectors were co-transfected into HEK293T cells using Lipofectamine LTX and cultured for 24 h. (**F**) The relative ratio of sensor vector to mutant vector in (**E**). Graphs show the mean ± SEM (*n* = 4; **A**,**C**,**E** and **F**) and (*n* = 3; B). (**A**–**C** and **F**) *P < 0.05; **P < 0.01; ***P < 0.001, Student’s *t*-test. Cohen’s effect size d in these data was >0.8.

To investigate whether the miR-19a-19b-20a sub-cluster regulates endogenous Tgfbr2 protein levels in primary lung fibroblasts, we retrovirally transduced the sub-cluster and the reporter gene ∆hLNGFR (hCD271) into *Col1a2*-GFP fibroblasts (Supplemental Fig. S1A). Transduction efficiencies were similar between the control and the miR-19a-19b-20a sub-cluster vectors, and >60% in all samples (Supplemental Fig. S1C). We observed that miR-19a, 19b and 20a-expression levels were significantly higher in hCD271^+^ fibroblasts transduced with the miR-19a-19b-20a sub-cluster-vectors (sub-cluster fibroblasts) as compared with those transduced with a control vector (control fibroblasts) (Fig. 3A). Because the half-life of *Tgfbr2* mRNA and Tgfbr2 protein was estimated to be between 1 and 5 h (average: 2–3 h)^22^, *Tgfbr2* mRNA and Tgfbr2 protein expression were measured at 5-h post-infection. We found that *Tgfbr2* mRNA expression did not change significantly (Fig. 3B); however, Tgfbr2 protein levels were significantly downregulated at 5-hour post-infection (Fig. 3C). To determine whether the miR-19a-19b-20a sub-cluster directly regulates Tgfbr2 protein levels, we assessed interactions between the miR-19a-19b-20a sub-cluster and the 3′ UTR of *Tgfbr2* mRNA. We found that the 3′ UTR of *Tgfbr2* mRNA contains 8-mer target sequences of each miRNA (Fig. 3D). A luciferase-reporter assay revealed that the miR-19a-19b-20a sub-cluster suppressed the expression of the wild-type *Tgfbr2* 3′ UTR reporter, but not that of the mutant *Tgfbr2* 3′ UTR reporters (Fig. 3E,F). These results suggested that the miR-19a-19b-20a sub-cluster suppressed Tgfbr2 protein levels by targeting the *Tgfbr2* 3′ UTR sequence in lung fibroblasts.

### miR-19a-19b-20a sub-cluster inhibits TGF-β1-mediated activation of primary lung fibroblasts

**Figure 4:**
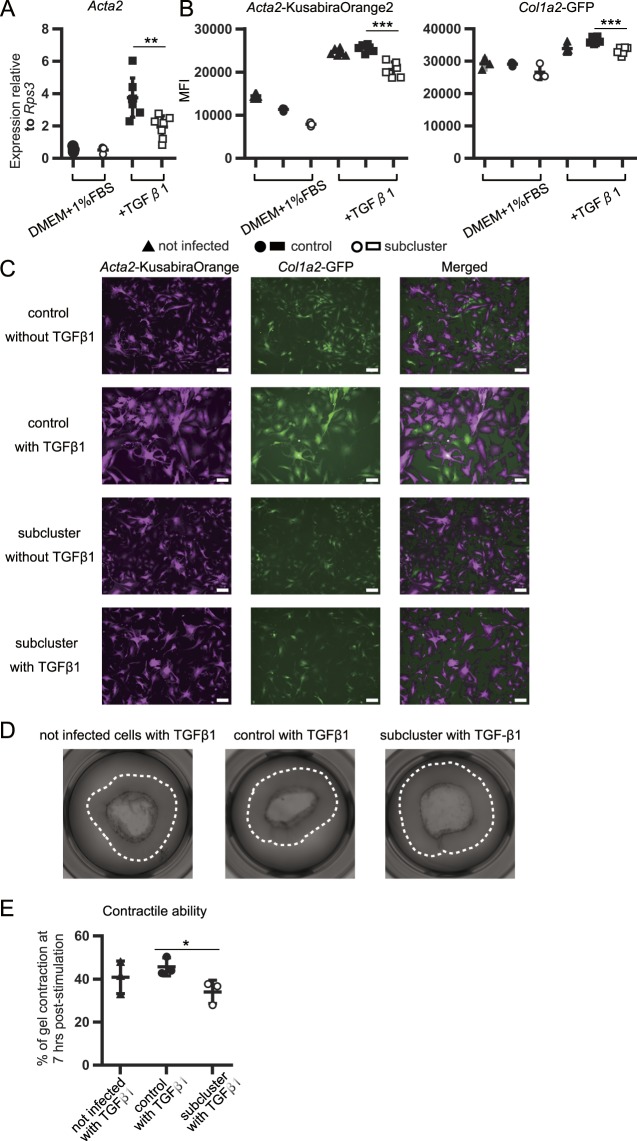
The miR-19a-19b-20a sub-cluster inhibits activation of primary lung fibroblasts *in vitro*. (**A–C**) Primary lung fibroblasts transduced by retrovirus were cultured with 1% FBS + DMEM for 12 h, followed by a change of medium to 1% FBS + DMEM in the presence or absence of TGF-β1. (**A**) Effect of the miR-19a-19b-20a sub-cluster on TGF-β1-mediated expression of *Acta2* mRNA in primary lung fibroblasts. qPCR measurement of mRNA expression after a 36-h stimulation with TGF-β1 (10 ng/mL). (**B**) Effect of the miR-19a-19b-20a sub-cluster on *Acta2*- and *Col1a2*-promoter activity in *Acta2*-Kusabira Orange2 × *Col1a2*-GFP fibroblasts under TGF-β1-stimulated conditions. Flow-cytometric analysis of the MFIs associated with *Acta2*-Kusabira Orange2 and *Col1a2*-GFP fibroblasts in hCD271^+^ cells after a 36-h stimulation with TGF-β1 (10 ng/mL). (**C**) Fluorescent images of *Acta2*-Kusabira Orange2 × *Col1a2*-GFP fibroblasts after a 36-h stimulation with TGF-β1 (10 ng/mL). *Acta2*-Kusabira Orange2 (magenta) and *Col1a2*-GFP (green). Scale bars: 100 μm. Representative images (*n* = 3) are shown. (**D**,**E**) Effect of the miR-19a-19b-20a sub-cluster in the contractile activity of primary lung fibroblasts in the presence or absence of TGF-β1 stimulation. *Col1a2*-GFP fibroblasts (3 × 10^4^) transduced by retrovirus were plated on collagen gels. After 2 h, the gels were mechanically loosened from the sides of the wells, and the cells were cultured in medium containing TGF-β1 (10 ng/mL) and 1% FBS + DMEM. The surface area of the collagen gel was measured using ImageJ at 0- and 7-h post-stimulation, and the percentage of gel contraction in each cell group was calculated. (**D**) Gel images at 7-h post-stimulation. Dashed line shows the size of the gels at the stimulation-start point. Representative images (*n* = 3) are shown. (**E**) Percentage of collagen gel contraction at 7-h post-stimulation. Graphs show the mean ± SEM (*n* = 7; **A**), (*n* = 6; **B**), and (*n* = 3; **E**). *P < 0.05; **P < 0.01; ***P < 0.001, Student’s *t*-test (**A**,**B**, and **E**). Cohen’s effect size d in these data was >1.8.

To assess the effect of the miR-19a-19b-20a sub-cluster on TGF-β1-mediated lung fibroblast activation, we measured *Acta2* expression and *Col1a2*-promoter activity, both major downstream targets of TGF-β1, in primary lung fibroblasts in the presence and absence of TGF-β1 stimulation. Because the half-life of *Acta2* mRNA is estimated at ~18 h, and the half-life of Acta2 protein is ~59 h^23^, the transduced fibroblasts were analyzed at 4-days post-infection. Flow-cytometric analysis revealed that the sub-cluster fibroblasts expressed significantly lower levels of α-SMA and *Col1a2*-GFP as compared with control fibroblasts (Supplemental Fig. S2A,B). When stimulated with TGF-β1 for 24 h after transduction, the sub-cluster fibroblasts expressed significantly lower levels of *Acta2* mRNA as compared with control fibroblasts (Fig. 4A). Similar results were obtained using primary lung fibroblasts from *Acta2*-Kusabira Orange2 × *Col1a2*-GFP mice transduced with the miR-19a-19b-20a sub-cluster (Fig. 4B,C).

We then evaluated the effect of the miR-19a-19b-20a sub-cluster on the TGF-β1-dependent contractile ability of lung fibroblasts using a collagen gel-contraction assay. In the presence of TGF-β1 stimulation, collagen gel-surface areas were larger in the sub-cluster fibroblasts as compared with control fibroblasts, suggesting that the miR-19a-19b-20a sub-cluster inhibited the TGF-β1-dependent contractile ability of lung fibroblasts (Fig. 4D,E). These results demonstrated that the miR-19a-19b-20a sub-cluster inhibited TGF-β1-induced upregulation of α-SMA expression, *Col1a2*-promoter activity, and contractile ability in lung fibroblasts.

### miR-19a-19b-20a sub-cluster regulates fibroblast activation in the fibrotic lung

**Figure 5:**
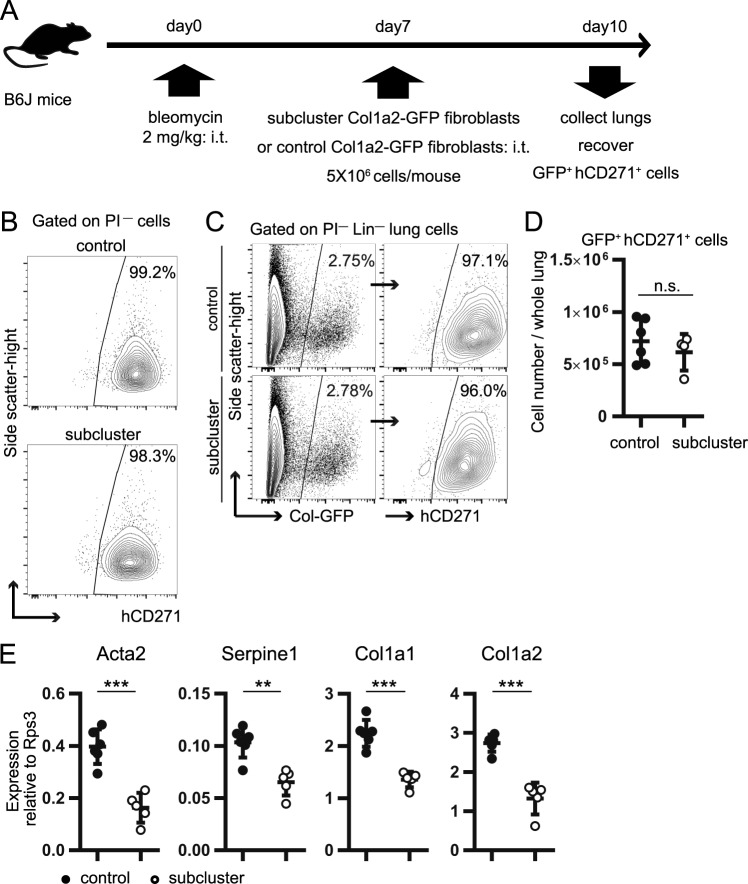
The miR-19a-19b-20a sub-cluster regulates fibroblast activation in the fibrotic lung in adoptively transferred models. (**A**) Scheme of intratracheal-transfer experiments. *Col1a2*-GFP fibroblasts were transduced by retrovirus and cultured with 10% FBS + DMEM for 3 days. hCD271^+^ cells were magnetically isolated, cultured for another 10 days, and passaged three times. The harvested cells (5 × 10^6^ cells/mouse) were intratracheally transferred to the lungs of B6J mice at day 7 post-bleomycin administration, and engrafted GFP^+^ hCD271^+^ cells were recovered at day 10 by cell sorting. (**B**) Flow-cytometry plots of control and miR-19a-19b-20a sub-cluster fibroblasts prior to intratracheal transfer. Transduction efficiency was >98%. (**C**) Flow-cytometry plots of whole-lung cells from intratracheally transferred mice at day 10 post-bleomycin administration. Transduction efficiency of GFP^+^ fibroblasts was >96%. (**D**) The number of engrafted GFP^+^ hCD271^+^ cells at day 10 post-bleomycin administration. (**E**) qPCR measurement of *Acta2* and *Serpine1* mRNA expression in GFP^+^ hCD271^+^ cells. (D and E) Graphs show the mean ± SEM [*n* = 6 (control) and *n* = 5 (sub-cluster)]. **P < 0.01; ***P < 0.001, Student’s *t*-test. Cohen’s effect size d in these data was >2.7. (**C,D**) Representative plots [*n* = 5 (control) and *n* = 4 (sub-cluster)] are shown.

To evaluate whether the miR-19a-19b-20a sub-cluster overexpression suppresses fibroblast activation in fibrotic lung, we transferred sub-cluster fibroblasts or control fibroblasts intratracheally into the lungs of mice at day 7 post-bleomycin administration. Because intratracheally transferred fibroblasts become incorporated into fibrotic lesions in the bleomycin-treated lung at 3-days post-transfer^24^, we analyzed transferred fibroblasts at this time point (Fig. 5A). Donor fibroblasts were identified by *Col1a2*-GFP expression, whereas gene-transduced donor fibroblasts were identified by hCD271 expression. The transduction efficiency for all samples before and after intratracheal transfer was >96% (Fig. 5B,C). The number of GFP^+^ hCD271^+^ cells in the lungs at 3-days post-transfer did not differ significantly between recipients of the sub-cluster fibroblasts and control fibroblasts (Fig. 5D). However, *Acta2, Serpine1, Col1a1*, and *Col1a2* mRNA-expression levels in sorted donor fibroblasts were significantly lower in the sub-cluster fibroblasts as compared with control fibroblasts, suggesting that the miR-19a-19b-20a sub-cluster partially inhibited the upregulation of fibrosis-prone genes in fibroblasts of fibrotic lung (Fig. 5E).

### miR-19a-19b-20a sub-cluster modulates the TGF-β-related changes in the global transcriptome profile of activated lung fibroblasts

**Figure 6:**
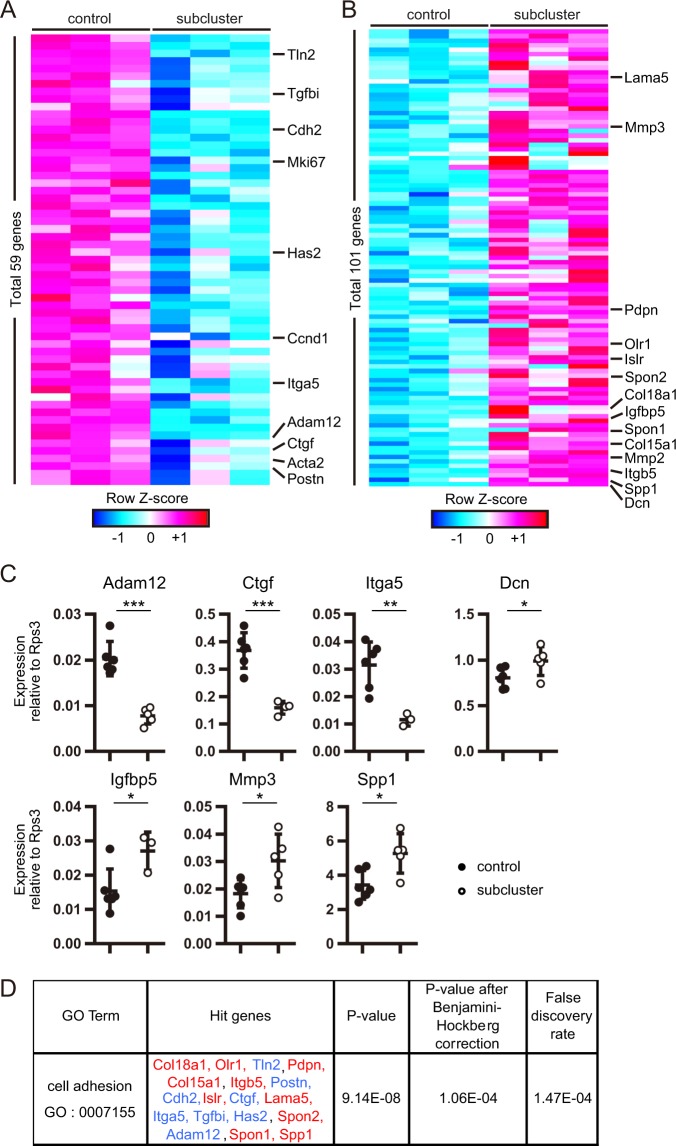
The miR-19a-19b-20a sub-cluster modulates the global transcriptome landscape of activated fibroblasts in fibrotic lungs. (**A**,**B**) Gene-expression profile of GFP^+^ hCD271^+^ intratracheally transferred lung fibroblasts at day 3 post-transfer. Intratracheal transfer was performed on day 7 post-bleomycin administration. Heat map represents the (**A**) downregulated and (**B**) upregulated genes in the sub-cluster-fibroblasts. Each column represents an miRNA group, and each row represents an individual gene. (**C**) qPCR validation of *Adam12*, *Ctgf*, *Itga5*, *Dcn*, *Igfbp5*, *Mmp3*, and *Spp1* expression in GFP^+^ hCD271^+^ intratracheally transferred lung fibroblasts at day 3 post-transfer. Intratracheal transfer was performed on day 7 post-bleomycin administration. (**D**) GO analysis of 160 differentially expressed genes using DAVID version 6.8. Significantly enriched GO terms and associated genes are shown. (**C**) Graphs show the mean ± SEM [*n* = 6 (control) and *n* = 5 (sub-cluster)]. *P < 0.05; **P < 0.01; ***P < 0.001, Student’s *t*-test. Cohen’s effect size d in these data was >1.3. See also Supplemental Table S5.

To determine how the miR-19a-19b-20a sub-cluster regulates the global gene-expression profile of activated lung fibroblasts, we performed 3′ SAGE-seq analysis on intratracheally transferred control or the sub-cluster fibroblasts at 3-days post-transfer in the bleomycin model. We identified 160 genes that were differentially expressed in the sub-cluster fibroblasts relative to control fibroblasts (59 upregulated and 101 downregulated) and exhibiting a >1.5-fold-change in expression (*P* < 0.05) (Fig. 6A,B; see Supplemental Table S5 for the full list of differentially expressed genes). The range of changes in gene expression was between 0.33- to 0.66-fold downregulation and 1.5- to 4.73-fold upregulation. The downregulated genes included TGF-β-associated pro-fibrotic genes, such as *Acta2*, *Adam12*^25^, *Ctgf*^3^, *Has2*^26^, *Itga5*^27^, *Postn*^28^, and *Tgfbi*^29^, and cell cycle-related genes, such as *Ccnd1* and *Mki67* (Fig. 6A). Additionally, *Serpine1* expression was significantly downregulated (0.76-fold) in the sub-cluster fibroblasts relative to control fibroblasts (Supplemental Table S5). By contrast, upregulated genes included anti-fibrotic genes, such as *Dcn*^30^, *Igfbp5*^31^, and *Mmp3*^32^ (Fig. 6B). Unexpectedly, some fibroblast-activation-associated genes such as *Spp1*^33^ and *Mmp2*^34^, were also upregulated. qPCR analysis confirmed the changes in gene expression for *Adam12*, *Dcn*, *Igfbp5*, *Mmp3* and *Spp1* (Fig. 6C). To investigate the biological functions modulated by the miR-19a-19b-20a sub-cluster overexpression in activated lung fibroblasts, we performed GO^35^ analysis on the 160 genes exhibiting the sub-cluster-mediated differential expression, with cell adhesion (GO:0007155) the only function significantly enriched among these genes (Fig. 6D). These results suggested that the miR-19a-19b-20a sub-cluster suppresses a part of the TGF-β-signaling-associated gene expression in the activated fibroblasts during bleomycin-induced lung fibrosis.

## Discussion

In this study, we identified miRNA signatures in lung fibroblasts of transient/progressive murine pulmonary fibrosis models. Among these signatures, we found that the miR-19a-19b-20a sub-cluster suppressed TGF-β-associated gene expression and primary lung fibroblast activation.

We used both bleomycin-induced transient and silica-induced progressive pulmonary fibrosis models to identify the fibrosis-associated common activation signature of miRNA expression in primary fibroblasts. Intratracheal instillation of bleomycin induces acute epithelial injury and transient fibrotic responses. Silica is ingested by macrophages, resulting in their activation and production of pro-fibrotic cytokines, such as tumor necrosis factor-α, PDGFs, and TGF-β, leading to progressive fibrotic responses. Notably, despite the differences in etiology between these two fibrosis models, most of the differentially expressed miRNAs (159 of 165 miRNAs) were commonly upregulated or downregulated in line with fibrosis progression in activated fibroblasts from both models. Additionally, pathway analysis suggested possible contributions of these miRNAs in attenuating the TGF-β-signaling pathway and fatty acid metabolism-related pathways. A previous study reported that lung-resident fibroblasts contain lipid droplets, and that lipogenic genes contribute to lung fibroblast activation^36^. Moreover, a meta-analysis of global-expression profiles of mRNA and miRNA data from IPF patients revealed that the expression pattern of IPF-associated miRNA signatures is associated with that of TGF-β-signaling-related and lipid-metabolism-related genes^37^. Our results and previous reports suggested that the identified common miRNA-expression signatures in activated fibroblasts constitute a fibroblast-activation marker in a variety of fibrotic interstitial lung diseases, including IPF.

Here, we identified the miR-19a-19b-20a sub-cluster, a component of the miR-17~92 cluster, as fibroblast-activation-associated miRNAs. A previous study reported that expression of miR-17~92 cluster components, including the miR-19a-19b-20a sub-cluster, decreased in lung tissues from IPF patients and those from mice treated with bleomycin during late phase (day 28)^10^. Because fibroblasts constitute 15% to 20% of the whole-cell population in lungs, especially after induction of fibrotic disease in mice^33,38^, miRNA expression in whole lung tissues observed in the previous study might not reflect that observed in fibroblasts. The differences in the miR-19a-19b-20a sub-cluster expression between whole lung tissues and purified fibroblasts might be due to a specific regulatory mechanism associated with the sub-cluster expression in fibroblasts during fibrotic responses.

miR-19a-19b target TGFBR2 in human cardiac fibroblasts^39^, and miR-20a inhibits TGF-β signaling by targeting TGFBR2 in non-fibroblastic cells, including cancer cells^40^, endothelial cells^41^. Consistent with previous studies, we observed that Tgfbr*2* expression was suppressed by the miR-19a-19b-20a sub-cluster in primary lung fibroblasts and associated with suppression of TGF-β1-associated fibroblast activation. Intratracheal transfer of the sub-cluster fibroblasts revealed that inhibition of TGF-β1-associated fibroblast activation by the miR-19a-19b-20a sub-cluster also occurred in activated fibroblasts from a fibrotic lung. Moreover, the expression of *Serpine1*, a major TGF-β–responsive gene^42^, decreased in the sub-cluster-fibroblasts from a fibrotic lung, supporting speculation that the miR-19a-19b-20a sub-cluster suppresses TGF-β1 signaling in lung fibroblasts during pulmonary fibrosis. Our pathway analysis and previous report also suggested a possible contribution of miR-17~92 cluster components in TGF-β1 signaling in lung fibroblasts^10^. Overexpression of the miR-17~92 cluster decreases the amount of actin cytoskeleton and the expression of CTGF^10^, a downstream target of TGF-β1 signaling^3^. Overexpression of miR-19a and miR-19b reduces CTGF expression in lung fibroblast cell lines^11^. These reports and our results suggested that miR-17~92 cluster components, including the miR-19a-19b-20a sub-cluster, possibly cooperatively inhibit TGF-β1-mediated lung fibroblast activation.

Transcriptome analysis of adoptively transferred fibroblasts revealed decreases in expression of the pro-fibrotic genes *Ctgf*, *Adam12*, and *Itga5* and increases in the expression of the anti-fibrotic genes *Dcn* (*Decorin*) and *Mmp3* among multiple genes affected by the miR-19a-19b-20a sub-cluster overexpression. CTGF is a pro-fibrotic matricellular protein that contributes to pulmonary fibrosis through transcriptional activation of COL1A2^43^. Overexpression of CTGF in fibroblasts results in tissue fibrosis in skin, lungs, and kidneys^44^, with CTGF induced by TGF-β capable of mediating the pro-fibrotic effects of TGF-β *in vitro*^3^. ADAM12 is a member of the ADAM protein family of disintegrins and metalloproteases, with Adam12^+^ cells reported as specific progenitors of collagen-overproducing cells generated following acute tissue injury. Genetic ablation of these Adam12^+^ cells limits generation of pro-fibrotic cells and interstitial collagen accumulation in muscle and dermis acute-injury models^45^. ITGA5 belongs to the integrin α-chain family, with ITGA5/ITGB1 expression facilitating lung stromal progenitor-cell differentiation into lung myofibroblasts^27^. Decorin is a proteoglycan that inhibits fibrotic responses by inhibiting TGF-β signaling^46^, and adenoviral Dcn transduction in lung tissues suppresses bleomycin-induced pulmonary fibrosis^30^. Collectively, the transcriptional changes associated with these genes support the anti-fibrotic roles related to the miR-19a-19b-20a sub-cluster expression in fibroblasts during pulmonary fibrosis.

We also observed upregulated *Spp1* expression in the sub-cluster fibroblasts. *Spp1* encodes osteopontin, an ECM protein previously reported as a marker of activated fibroblasts present at the site of tissue remodeling in bleomycin-induced pulmonary fibrosis. Importantly, osteopontin^+^ and α-SMA^+^ fibroblasts show different distribution patterns in bleomycin-induced fibrotic lesions^33^. Additionally, osteopontin expression is induced by PDGF signaling in smooth-muscle cells^47^, which is another pro-fibrotic pathway distinct from TGF-β signaling. Therefore, it is possible that the sub-cluster suppresses only a subset of fibroblast-activation pathways, including that related to TGF-β signaling. Overall, we propose that upregulation of the miR-19a-19b-20a sub-cluster represents a compensatory response in activated fibroblasts that suppress detrimental TGF-β-associated fibrogenic activation of lung fibroblasts following tissue injury.

The progress and pathology of IPF differ from murine pulmonary fibrosis models; however, one commonality involves TGF-β activation of both human and murine lung fibroblasts. Considering that miR-19a-3p, 19b-3p and 20a-5p are conserved in mice and humans, the knowledge provided in this study will likely be applicable to IPF-patient-derived fibroblasts. Development of therapeutic strategies for the fibroblast-specific control of the miR-19a-19b-20a sub-cluster expression might lead to the identification of new therapeutic approaches for treating fibrotic diseases, including IPF.

## Materials and Methods

### Mice

C57BL/6J (B6) female mice were purchased from Japan SLC (Hamamatsu, Japan) or CLEA Japan (Tokyo, Japan). *Col1a2*-GFP female mice (C57BL/6 background for >10 generations) were generated in previous study^48^. *Acta2*-Kusabira Orange2 mice were generated as described in the Methods section of the online supplement. *Acta2*-Kusabira Orange2 × *Col1a2*-GFP male mice were generated by cross-breeding *Col1a2*-GFP mice and *Acta2*-Kusabira Orange2 mice. Experiments were initiated when mice were 6- to 12-weeks old. Mice were bred and maintained in specific pathogen-free facilities at the University of Tokyo, were group-housed with freely available food and water, and were under standard conditions with a light-dark cycle of 12 hours light and 12 hours dark. All methods were carried out in accordance with relevant guidelines of the University of Tokyo, and all experimental protocols were approved by the University of Tokyo.

### Bleomycin- or silica-induced pulmonary fibrosis

Intratracheal instillation of bleomycin and silica was performed as described previously^38^.

### Preparation of primary lung fibroblasts

Primary lung fibroblasts were isolated from *Col1a2*-GFP mice (*Col1a2*-GFP fibroblasts) and *Acta2*-Kusabira Orange2-*Col1a2*-GFP mice (*Acta2*-Kusabira Orange2-*Col1a2*-GFP fibroblasts) as described in the Methods section of the online supplement.

### Global miRNA-expression profiling

Small RNAs were isolated from lineage^−^ GFP^+^ lung fibroblasts using a mirVana miRNA-isolation kit (Thermo Fisher Scientific, Waltham, MA, USA). Small RNA libraries were constructed using an Ion Total RNA-seq kit v2 (Thermo Fisher Scientific) according to manufacturer instructions. The samples were sequenced twice on an Ion PGM system using an Ion PGM sequencing 200 kit (Thermo Fisher Scientific) according to manufacturer instructions. CLC Genomics Workbench software version 6.0.5 (CLC Bio, Aarhus, Denmark) was used to trim the sequence reads. miRNA expression analysis was performed by counting the resulting tags using miRBase database^49^ (v19; http://www.mirbase.org/). Total tag counts were normalized to 80,000 tags, and the tag counts from two independent sequencing reads were averaged. Raw data from these experiments were deposited in the NCBI Gene Expression Omnibus (GEO; http://www.ncbi.nlm.nih.gov/geo; accession GSE100115).

### Grouping of global miRNA-expression data

To group global miRNA-expression data, miRNAs with maximum tag counts (<10) across all experiments were filtered out. We then selected miRNAs exhibiting a fold-change of ≥2 between at least two samples. The total tag count for the 165 selected miRNAs was log_2_(X + 1) transformed, and miRNAs were grouped using R-3.3.1 software (https://cran.r-project.org/) and the WGCNA package^20^ (power = 8, merge_thre = 0.25). Each detected miRNA group was further divided into positive- and negative-correlated groups using the CLICK method^21^. The expression level of each group was Z-scaled and visualized using R-3.3.1 software (https://cran.r-project.org/).

### Kyoto Encyclopedia of Genes and Genomes (KEGG) pathway analysis and detection of miRNA-gene interactions for each miRNA group

KEGG pathway analysis of miRNA-target genes was performed on each miRNA group using DIANA-miRPath version 3.0 software^50^. Prediction of miRNA-target genes was based on results of Tarbase version 7.0^50^. Because of a restriction on the number of mature miRNAs capable of being analyzed using this software, miRNAs with average tag counts (<10) across all experiments were filtered out. KEGG pathway terms with false-detection-rate-corrected *P*-values < 0.01 were selected as highly enriched pathway terms in each miRNA group. Predicted miRNA-gene interactions in each KEGG pathway were simultaneously detected by DIANA-miRPath version 3.0 software using Tarbase version 7.0 as the database.

### Quantitative real-time polymerase chain reaction (qPCR)

Total RNA from transduced lung fibroblasts was isolated using TRIzol reagent (Thermo Fisher Scientific). qPCR of mRNA was performed as described previously^38^. miRNA were reverse transcribed using TaqMan MicroRNA assays (Thermo Fisher Scientific) according to manufacturer instructions. qPCR analysis of miRNA was performed using THUNDERBIRD Probe qPCR mix (Toyobo) on an ABI 7500 real-time PCR system (Thermo Fisher Scientific). Primer sequences are listed in Table 1. Probe IDs for qPCR analyses of miRNAs were as follows (miR-19a: 000395; miR-19b: 000396; miR-20a: 000580; miR-23a: 000399). Relative gene-expression levels of mRNA and miRNA were calculated after normalization against the expression of the reference genes Rps3 and miR-23a.

**Table 1:** Primer sequences used for qPCR.

Genes	Forward primers (5′ to 3′)	Reverse primers (5′ to 3′)
*Acta2*	TCGGATACTTCAGCGTCAGGA	GTCCCAGACATCAGGGAGTAA
*Adam12*	CACACGGATCATTGTTACTACCA	ATTGGCTCTAAGCTGTACGTTTT
*Col1a1*	AGACATGTTCAGCTTTGTGGAC	GCAGCTGACTTCAGGGATG
*Col1a2*	GGTGAGCCTGGTCAAACGG	ACTGTGTCCTTTCACGCCTTT
*Ctgf*	CTGCAGACTGGAGAAGCAGA	GCTTGGCGATTTTAGGTGTC
*Dcn*	GAGGGAACTCCACTTGGACA	TTGTTGTTGTGAAGGTAGACGAC
*Itga5*	CTTCTCCGTGGAGTTTTACCG	GCTGTCAAATTGAATGGTGGTG
*Mmp3*	ACATGGAGACTTTGTCCCTTTTG	TTGGCTGAGTGGTAGAGTCCC
*Rps3*	CGGTGCAGATTTCCAAGAAG	GGACTTCAACTCCAGAGTAGCC
*Serpine1*	GGCACCTTTGAATACTCAGGA	TTTCCCAGAGACCAGAACCA
*Spp1*	GGAGGAAACCAGCCAAGG	TGCCAGAATCAGTCACTTTCAC
*Tgfbr2*	CCGCTGGAACATATCGTCCTGTG	AGTGGATGGATGGTCCTATTACA

### Retroviral miRNA transduction

A 479 bp fragment containing miR-19a, miR-19b and miR-20a precursors was amplified by nested PCR by using PrimeSTAR Max DNA polymerase (Takara) and cloned into the downstream region of the CMV promoter in a modified pMY retroviral vector containing a ∆hLNGFR/hCD271 reporter gene (a truncated form of low-affinity nerve-growth factor receptor) (Supplemental Fig. S1A). Since the design of expression vector for individual miRNAs in miR-19a-19b-20a sub-clusters was technically difficult, we were unable to construct these vectors. Primer sequences for nested PCR are shown in Supplemental Fig. S1B. miRNA expression was confirmed using a sensor vector derived from the psiCHECK-2 vector (Promega, Madison, WI, USA). Retroviral vectors were produced in GP2-293T cells as described previously^51^. Viral supernatant was passed through 0.45-μm filters and concentrated using Amicon Ultra-15 centrifugal filters (50-kDa MWCO; Millipore, Bedford, MA, USA). The viral supernatant was concentrated to a pellet using a lentiX-concentrator (Takara, Tokyo, Japan) according to manufacturer instructions, and the pellet was dissolved with 10% FBS + DMEM. For miRNA transduction, 1 × 10^4^ primary lung fibroblasts were transferred to 48-well plates. After a 12-h incubation, the cells were exposed to the viral supernatant (diluted to appropriate density) for 24 h.

### Flow cytometry

Flow-cytometric analyses were performed as described previously^38^. Cells were stained with allophycocyanin (APC)- or phycoerythrin (PE)-conjugated anti-hCD271 antibody (clone ME20.4; BioLegend, San Diego, CA, USA) or anti-Tgfbr2 antibody (goat polyclonal, catalog no. FAB532A; BioLegend) for 30 min at 4 °C. We minimized compensation by choosing fluorescent combinations to measure mean fluorescence intensity (MFI). Intracellular α-SMA staining were performed as described previously^24^. Cells were stained with the APC-conjugated anti-α-SMA antibody (clone 1A4; R&D Systems, Minneapolis, MN, USA) for 30 min at 4 °C.

### Luciferase assay

Sensor and mutant vectors for Tgfbr2 were created from the psiCHECK-2 vector (Promega). Each of the control and sub-cluster vectors, and each of the sensor and mutant vectors were co-transfected into HEK293T cells using Lipofectamine LTX (Thermo Fisher Scientific) and cultured for 24 h. Renilla luciferase–firefly luciferase ratios in the cells were measured using a Dual-Glo reporter assay system (Promega) according to manufacturer instructions.

### Cell culture under TGF-β1-stimulated conditions

Lung fibroblasts were cultured with 1% FBS + DMEM for 12 h, followed by a medium change to 1% FBS + DMEM in the presence or absence of TGF-β1 (10 ng/mL, R&D Systems). In some experiments, images of cultured fibroblasts were captured by a BZ-X700 fluorescence microscope (Keyence, Osaka, Japan).

### Collagen gel contraction assay

Collagen gels were prepared according to manufacturer instructions to a final collagen concentration of 1.5 mg/mL (rat tail Col1; BD Biosciences). After transduction, 3 × 10^4^ *Col1a2*-GFP lung fibroblasts were plated on the collagen gels in a 48-well plate. After cell attachment to the collagen gels (2 h), the gels were mechanically loosened from the sides of the wells, and the cells were cultured with 1% FBS + DMEM in the presence or absence of TGF-β1. The relative percentage of gel contraction represented the gel surface area covered each hour divided by the surface area just after mechanical loosening. The collagen gel images were quantified using ImageJ version 1.47t (http://imagej.nih.gov/ij; National Institutes of Health, Bethesda, MD).

### Intratracheal transfer of lung fibroblasts

Intratracheal transfer was performed as described previously^24^. Details are provided in the Methods section of the online supplement. Since adoptively transferred fibroblasts were efficiently integrated into the fibrotic foci of bleomycin model, but not of silica model, we performed this experiment only in bleomycin model.

### Amplification of the whole transcript of fibroblasts

The whole transcript of intratracheally transferred fibroblasts was amplified according to a previous report, with some modifications^52^. Details are provided in the Methods section of the online supplement. Primer sequences are shown in Supplemental Table S1.

### 3′ serial analysis of gene expression (3′SAGE)-library generation and sequencing

Generation of Na SAGE library was performed according to a previous report, with some modifications^53^. Details are provided in the Methods section of the online supplement. Raw data from these experiments were deposited in NCBI GEO (http://www.ncbi.nlm.nih.gov/geo; accession GSE100116). Primer and adapter sequences are shown in Supplemental Table S2.

### Analysis of 3′SAGE-seq data

Analysis of 3′SAGE-seq data was performed according to a previous report, with some modifications^54–57^. Details are provided in the Methods section of the online supplement. Genes exhibiting differential-expression levels associated with an adjusted *P*-value < 0.05, a fold change >1.5, and a minimum expression level >30 were identified as showing statistically significant differential expression. Gene ontology (GO) analysis of the differentially expressed genes was performed by using DAVID version 6.8^58^.

### Statistical analyses

Statistical comparisons were performed using unpaired Student’s *t*-tests (two-tailed), one-way analysis of variance (ANOVA) with Tukey-Kramer’s multiple-comparison post hoc tests, and a likelihood ratio test with Benjamini-Hockberg correction (transcriptome analysis). A *P* < 0.05 was considered statistically significant. Data are expressed as the mean ± standard error of the mean (SEM). Effect size was measured with Cohen’s d. Statistical analysis was performed using Prism software version 5.01 (GraphPad Software, La Jolla, CA, USA) or R-3.3.1 software (https://cran.r-project.org/).

## Electronic supplementary material

Supplemental Information and Figures

Supplementary tables
