# Supplementary material for: Lung fibroblasts express a miR-19a-19b-20a sub-cluster to suppress TGF-β-associated fibroblast activation in murine pulmonary fibrosis
Source: Sci Rep. 2018 Nov 9;8:16642. doi: 10.1038/s41598-018-34839-0 (PMC6226532; doi:10.1038/s41598-018-34839-0)
Supplement: Supplementary file 1 — Supplemental Information and Figures [file 41598_2018_34839_MOESM1_ESM.pdf]

Lung fibroblasts express a miR-19a-19b-20a subcluster to suppress TGF-β-associated fibroblast activation in murine pulmonary fibrosis

Kunihiko Soma[1,2,3,*], Shigeyuki Shichino[1,3,*], Shin-ichi Hashimoto[1,3,4], Satoshi Ueha[1,3], Tatsuya Tsukui[1], Takuya Nakajima[1,3], Hiroshi I Suzuki[5], Francis HW Shand[1], Yutaka Inagaki[6], Takahide Nagase[2] and Kouji Matsushima[1,3,†]

[1]Department of Molecular Preventive Medicine, Graduate School of Medicine, The University of Tokyo, Tokyo, Japan.

[2]Department of Respiratory Medicine, Graduate School of Medicine, The University of Tokyo, Tokyo, Japan.

[3] Division of Molecular Regulation of Inflammatory and Immune Diseases, Research Institute of Biomedical Sciences, Tokyo University of Science, Chiba, Japan.

[4]Division of Nephrology, Department of Laboratory Medicine, Kanazawa University, Ishikawa, Japan.

[5]David H. Koch Institute for Integrative Cancer Research, Massachusetts Institute of Technology, MA, USA.

[6]Center for Matrix Biology and Medicine, Graduate School of Medicine, Tokai

University, Kanagawa, Japan.

* These authors contributed equally to this work

† Correspondence to: Satoshi Ueha, PhD

Associate Professor

Division of Molecular Regulation of Inflammatory and Immune Diseases

Research Institute of Biomedical Sciences

Tokyo University of Science

2669 Yamasaki, Noda, Chiba 278-0022, Japan

Phone: +81-4-7121-4116

E-mail: ueha@rs.tus.ac.jp

Supplemental Information (MATERIALS AND METHODS)

Acta2-Kusabira Orange 2 (KO2) mice

We crossbred *Col1a2*-minimum-promoter/enhancer-ΔhLNGFR (hCD271)-CreER-poly(A)-Acta2 promoter-loxP-poly(A)-loxP-KO2 BAC transgenic mice (T. Tsukui; details to be published elsewhere) and ROSA26-CAG promoter-CreER transgenic mice purchased from Jackson Laboratory (stock number: 004682; Bar Harbor, ME, USA) and treated double-heterozygote mice with tamoxifen to enable germ-line recombination of the loxP sequence. Confirmation of successful germ-line recombination of the loxP sequence was verified with localization of Acta2 promoter-KO2 on the Y-chromosome and correlation of KO2 expression with Acta2 expression, with hCD271 expression undetectable.

Preparation of primary lung fibroblasts

Lungs were cut into small pieces with a razor blade and digested enzymatically using 0.2% collagenase (Wako, Osaka, Japan), 0.96 mg/mL dispase II (Roche, Basel, Switzerland), and 20 kU/mL DNase I (Sigma-Aldrich, St. Louis, MO, USA) for 60 min at 37°C. Erythrocytes and silica particles were removed by 70% percoll (GE Healthcare,

Buckinghamshire, UK) density separation. The single-cell suspensions were then depleted of lineage^{+} (CD31^{+}, CD45^{+}, CD146^{+}, EpCAM^{+}, and Ter119^{+}) cells by negative selection with an AutoMACS cell separator (Miltenyi Biotech, Bergisch Gladbach, Germany). For global miRNA-expression profiling, lineage^{-} (CD31^{-}, CD45^{-}, EpCAM^{-}, and Ter119^{-}) GFP^{+} fibroblasts were further purified by cell sorting using a FACS Aria II system (BD Biosciences, San Jose, CA, USA). For transcriptome analysis, lineage^{-} (CD31^{-}, CD45^{-}, CD146^{-}, EpCAM^{-}, and Ter119^{-}) GFP^{+} fibroblasts were further purified by cell sorting using a FACS Aria II system (BD Biosciences). For *in vitro* experiments, lineage^{-} cells were cultured in 10% fetal bovine serum (FBS)+Dulbecco's modified Eagle medium (DMEM) for >3 days, and nonadherent cells were removed from the culture. The resulting cells routinely consisted of >95% lineage^{-} GFP^{+} fibroblasts.

Intratracheal transfer of lung fibroblasts

Col1a2-GFP fibroblasts were transduced by retrovirus and cultured with 10% FBS+DMEM for 3 days. After positive selection of hCD271+ cells with an AutoMACS cell separator (Miltenyi Biotech), the cells were cultured for another 10 days with three

passages over a 3-day interval. Cell suspensions were administered to B6 mice at day 7 post-administration of 2 mg/kg bleomycin, with 5×10^6 cells transferred per mouse. Flow-cytometric analysis of recipient mouse lungs was performed at 3-days post-intratracheal-transfer. The lineage− GFP+ hCD271+ donor fibroblasts (10,000 cells) were simultaneously recovered from lungs by direct sorting in 500 µL of cell lysis buffer [1% LiDS, 100 mM Tris-HCl (pH 7.5), 500 mM LiCl, 10 mM ethylenediaminetetraacetic acid (EDTA), and 5 mM dithiothreitol (DTT)] and stored at −80ºC until further qPCR and transcriptome analyses.

Amplification of the whole transcript of fibroblasts

0.5 pmol of biotin-TEG-adapter-dT25 primers was bound to 20 µL of Dynabeads M270 streptavidin (Thermo Fisher Scientific). The washed beads (20 uL) were added to each cell lysis buffer containing 10,000 sorted fibroblasts and incubated for 30 min at room temperature with gentle rotation. Beads were washed once with wash buffer A [0.1% LiDS, 10 mM Tris-HCl (pH 7.5), 150 mM LiCl, and 1 mM EDTA] and three times with wash buffer B [10 mM Tris-HCl (pH 7.5), 150 mM LiCl, and 1 mM EDTA]. Beads were then suspended in 10 µL of RT mix 1 [1× SSIV buffer (Thermo Fisher Scientific), 2 mM dNTP, 2 M betaine (Sigma-Aldrich), 12 mM $MgCl_{2,}$ and 3.2 U/µL RNaseIn Plus

(Promega)] and incubated for 90 s at 70ºC, 5 min at 35ºC, and immediately cooled on ice. RT mix 2 [10 μL; 1× SSIV buffer, 10 mM DTT (Thermo Fisher Scientific), 10 U/μL Superscript IV (Thermo Fisher Scientific), and 2 M betaine (Sigma-Aldrich)] was added, and reverse transcription was performed for 5 min at 35ºC and 15 min at 50ºC. Beads were washed once with cell lysis buffer, twice with B&W-T buffer [5 mM Tris-HCl (pH 7.5), 1 M NaCl, 0.5 mM EDTA, and 0.1% Tween-20], once with Tris-HCl (pH 8.0) and 20 μL of RNase H mix [1× first-strand buffer (Life Technologies, Carlsbad, CA, USA), 5 mM DTT, 0.6 U RNase H (Thermo Fisher Scientific)], and incubated for 20 min at 37ºC to digest reverse-transcribed mRNA. Beads were washed, 20 μL of TdT mix [50 mM Tris-HCl (pH 8.0), 100 mM KCl, 3 mM $MgCl_2$, 1 mM $CoCl_2$ (Roche), 0.65 mM dATP (Thermo Fisher Scientific), and 15.2 U/μL TdT (Roche)] was added on an ice-chilled aluminum rack, and polyA-tailing was performed for 3 min 20 s at 37ºC. The reaction was stopped by adding 5 μL of 0.5 M EDTA, and the enzyme was heat-inactivated by incubation for 10 min at 65ºC. Beads were washed, 20 μL of second-strand synthesis mix [1× KAPA Hifi ReadyMix (KAPA Biosystems, Wilmington, MA, USA) and 0.4 μM anchored tagging primer] was added, and second-strand synthesis was performed according to the following program: 95ºC for 2 min, 98ºC for 20 s, 44ºC for 2 min, 72ºC for 7 min, and hold at 4ºC. Beads were washed,

and 1/4 of the beads were used for the first round of whole-transcript amplification (WTA) in 25 μL of first-round WTA mix [1× KAPA Hifi ReadyMix (KAPA Biosystems), 0.4 μM anchored tagging primer, and 0.4 μM 3′ WTA primer] using the following program: 95ºC for 3 min, seven cycles of 98ºC for 20 s, 65ºC for 15 s, and 72ºC for 7 min, followed by 72ºC for 5 min and a hold at 4ºC. PCR products were purified twice with 0.6× AmPure XP beads (Beckman Coulter) and eluted with 23 μL of nuclease-free water. Second-round WTA mix [27 μL; 1× KAPA Hifi ReadyMix (KAPA Biosystems), 0.614 μM 5′ WTA primer, and 0.614 μM Biotin-TEG-3′ WTA primer] was added, and the second round of WTA was performed using the following program: 95ºC for 3 min, nine cycles of 98ºC for 20 s, 65ºC for 15 s, and 72ºC for 7 min, followed by 72ºC for 5 min and a hold at 4ºC. PCR products were purified once with 0.6× AmPure XP beads and eluted with 25 μL of Tris-HCl (pH 8.0). Amplified whole transcripts were quantified using a Nanodrop 1000 (Thermo Fisher Scientific), and size distribution was analyzed by agarose electrophoresis and SYBR Gold staining (Thermo Fisher Scientific).

3’ serial analysis of gene expression (3’SAGE)-library generation and sequencing

100 ng of the whole-transcript library was restriction digested with *Nla*III (New England Biolabs, Ipswich, MA, USA) for 2 h at 37ºC, and biotinylated 3′-tail transcripts were immobilized on Dynabead M-280 streptavidin beads (Thermo Fisher Scientific). Beads were washed, and 10 pmol of the CS1-*Eco*P15I-*Nla*III adapter was ligated using a DNA ligation kit (Mighty Mix; Takara) for 30 min at 16ºC. Beads were washed three times with B&W-T buffer, once with 10 mM Tris-HCl (pH 8.0), suspended in 200 µL of *Eco*P15I digestion mix [1× NEBuffer 3.1, 1 mM ATP, and 0.2 U *Eco*P15I (New England Biolabs)], and digested for 16 h at 37ºC in a tightly sealed screw-cap tube with gentle rotation. Supernatant was purified using a Nucleospin Gel&PCR clean-up kit (Takara) and eluted twice with 12.5 µL of nuclease-free water. End-repair/polyA-tailing/ligation reactions were performed using NEBNext Ultra II modules (New England Biolabs) and 1.875 pmol of CS2-adapter according to manufacturer instructions. Reaction products were purified using a Qiagen MinElute Column (Qiagen, Hilden, Germany) and eluted with 13 µL of nuclease-free water. Barcoding mix [14.25 µL; 1× KAPA Hifi ReadyMix (KAPA Biosystems), 0.614 µM IonA-BC[N]-CS1-primer, and 0.614 µM Ion-trP1-CS2 primer] was added into 10.75 µL of elutant, and PCR enrichment was performed using

the following program: 98ºC for 45 s, nine cycles of 98ºC for 15 s, 65ºC for 30 s, and 72ºC for 90 s, followed by 72ºC for 1 min and a hold at 4ºC. Reaction products were purified using double-size selection of AmPure XP beads (0.8× → 0.8×) and eluted with 10 μL of Tris-HCl (pH 8.0). The size distribution of each library was analyzed using an Agilent DNA high-sensitivity kit (Agilent Technologies, Santa Clara, CA, USA), and library concentration was quantified using a KAPA library quantification kit for Ion Torrent (KAPA Biosystems). Each SAGE library was pooled into equimolar volumes, and the concentrations were adjusted to 100 pM. Sequencing was performed using an Ion Hi-Q chef kit, an Ion PI v3 chip kit, and an Ion Proton sequencer (Thermo Fisher Scientific) according to manufacturer instructions, except for alterations in the input-library concentration (100 pM) and cycle number (200).

Analysis of 3’SAGE-seq data

Adapter trimming and quality filtering of sequencing data were performed using Trimommatic-v0.36[1] and PRINSEQ-0.20.4[2]. Filtered reads were mapped to Refseq mm10 by using Bowtie2-2.2.5[3] with the following parameters: -t -p 11 -N 1 -D 200 -R 20 -L 20 -i S,1,0.50 --norc. Reads that were not mapped to *Nla*III sites were removed, and quantified tag numbers for each gene were used to represent the gene-expression

level. Total tag number was adjusted to 1 million tags, and between-sample normalization was performed using R-3.3.1 software (https://cran.r-project.org/) and the TCC package[4]. Differential-expression analysis between the miRNA subcluster-overexpressing group and the control group was performed using R-3.3.1 software (https://cran.r-project.org/) and the glmLRT formula with Benjamini-Hockberg correction of the EdgeR package[5].

References

1 Bolger, A. M., Lohse, M. & Usadel, B. Trimmomatic: a flexible trimmer for Illumina sequence data. *Bioinformatics* **30**, 2114-2120, doi:10.1093/bioinformatics/btu170 (2014).

2 Schmieder, R. & Edwards, R. Quality control and preprocessing of metagenomic datasets. *Bioinformatics* **27**, 863-864, doi:10.1093/bioinformatics/btr026 (2011).

3 Langmead, B. & Salzberg, S. L. Fast gapped-read alignment with Bowtie 2. *Nature Methods* **9**, 357-U354, doi:10.1038/nmeth.1923 (2012).

4 Sun, J., Nishiyama, T., Shimizu, K. & Kadota, K. TCC: an R package for comparing tag count data with robust normalization strategies. *Bmc Bioinformatics* **14**, doi:10.1186/1471-2105-14-219 (2013).

5 McCarthy, D. J., Chen, Y. & Smyth, G. K. Differential expression analysis of multifactor RNA-Seq experiments with respect to biological variation. *Nucleic Acids Research* **40**, 4288-4297, doi:10.1093/nar/gks042 (2012).

Supplemental Figure S1

A

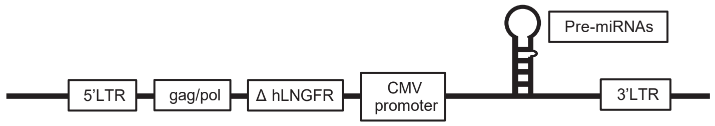

B

miR-19a-19b-20a first primer F	GGAAATCGGCATCTTCAGC
miR-19a-19b-20a first primer R	CTTTTTGTTCTAAGGTGCATCTAGTG
miR-19a-19b-20a second primer F	TATAAGCAGGCGGCCATGTAGGGGTCTCTCCATAGTTGTG
miR-19a-19b-20a second primer R	ACTGTGCTGGCGGCCGCAAATCCCAACCTGTGTAGAAAGG

C

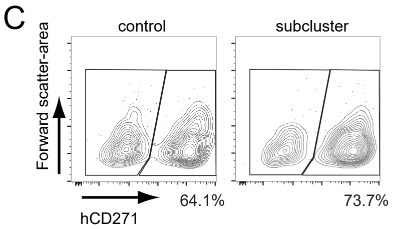

Supplemental Figure S1.
(A) Plasmid construction. miRNA precursors were cloned into the downstream of CMV promoter of modified pMY retroviral vector with ΔLNGFR/hCD271 (truncated form of low-affinity nerve growth factor receptor) reporter gene. (B) Primer lists for cloning the miR-19a-19b-20a subcluster (C) Flow-cytometry plots of control- or subcluster-vector transduced cells.

Supplemental Figure S2

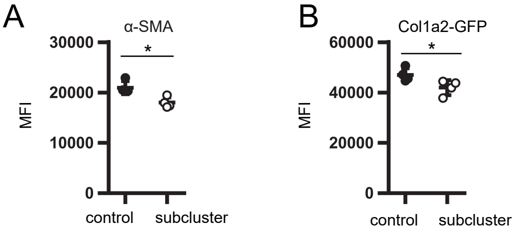

Supplemental Figure S2.
Effect of miR-19a-19b-20a subcluster in α-SMA expression and Col1a2 promoter activity without TGF-β1 stimulation. (A and B) Col1a2-GFP fibroblasts were transduced by retrovirus and cultured with 10% FBS+DMEM for 4 days. The cell were harvested and stained by PE anti-hCD271 antibody and APC anti-α-SMA antibody. The MFIs of APC and Col1a2-GFP in hCD271^{+} cells were measured by flow cytometry. α-SMA **(B)** *Col1a2*-GFP. Graphs show mean ± SEM of n=4. **P* < 0.05 by Student´s t-test **(A and B).** Cohen's effect size d of these data was >1.8.
